# Prevalence and assemblage identified of *Giardia duodenalis* in zoo and farmed Asiatic black bears (*Ursus thibetanus*) from the Heilongjiang and Fujian Provinces of China[Fn FN1]

**DOI:** 10.1051/parasite/2024048

**Published:** 2024-08-29

**Authors:** Jiani Chen, Liyuting Zhou, Wenjie Cao, Junchen Xu, Kuai Yu, Ting Zhang, Yiqing Wang, Jiayan Wang, Huicong Huang, Wei Zhao

**Affiliations:** School of Basic Medical Sciences, Wenzhou Medical University Wenzhou Zhejiang 325035 PR China

**Keywords:** *Giardia duodenalis*, Bear, assemblages, zoonotic, China

## Abstract

Captive and free-living wildlife serve as significant hosts for *Giardia duodenalis*. Asiatic black bears, valued for their economic and medicinal importance, are extensively farmed in China and also prevalent in zoos. However, studies on *G. duodenalis* in these animals in China are limited. Here, 218 feces samples of Asiatic black bears were collected: 36 from a zoo in Heilongjiang Province, and 182 from a farm in Fujian Province. Nested PCR of the *SSU rRNA* gene, followed by sequencing, was employed to determine the frequency and assemblage distribution of *G. duodenalis*. Positive samples underwent further analysis through multilocus genotyping (MLG) by amplifying the genes for glutamate dehydrogenase (*gdh*), β-giardin (*bg*), and triosephosphate isomerase (*tpi*). Of the 218 samples, *G. duodenalis* was detected in 22 cases at the *SSU rRNA* gene locus, including three from Heilongjiang and 19 from Fujian. Three assemblages were identified: A (*n* = 1), B (*n* = 16), and E (*n* = 2) in Fujian; and B (*n* = 3) in Heilongjiang. Out of the 22 positive samples, 20, 19, and 9 were effectively amplified and sequenced across the *tpi*, *gdh*, and *bg* loci, respectively. Seven samples were genotyped successfully at all three loci, identifying MLG-B1 (*n* = 1), MLG-B2 (*n* = 1), and MLG-B3 (*n* = 1), MLG-B4 (*n* = 1), MLG-B5 (*n* = 2), and MLG-B6 (*n* = 1) as the six assemblage B MLGs. This study marks the first documentation of *G. duodenalis* in Asiatic black bears in captivity in Fujian and Heilongjiang. The identification of zoonotic assemblages A and B, along with E, underscores potential public health concerns.

## Abstract (fr)

Les faunes captive et libre incluent des hôtes importants pour *Giardia duodenalis*. Les ours noirs d’Asie, appréciés pour leur importance économique et médicinale, sont couramment élevés en Chine et répandus dans les zoos. Cependant, les études sur *G. duodenalis* chez ces animaux en Chine sont limitées. Ici, 218 échantillons d’excréments d’ours noirs d’Asie ont été collectés, 36 dans un zoo de la province du Heilongjiang et 182 dans une ferme de la province du Fujian. La PCR imbriquée de l’ARNr SSU, suivie d’un séquençage, a été utilisée pour déterminer la fréquence et la distribution des assemblages de *G. duodenalis*. Les échantillons positifs ont subi une analyse plus approfondie par génotypage multilocus (MLG) en amplifiant les gènes de la glutamate déshydrogénase (*gdh*), de la β-giardine (*bg*) et de la triosephosphate isomérase (*tpi*). Sur les 218 échantillons, *G. duodenalis* a été détecté dans 22 cas par le locus du gène de l’ARNr SSU, dont trois du Heilongjiang et 19 du Fujian. Trois assemblages ont été identifiés : A (*n* = 1), B (*n* = 16) et E (*n* = 2) dans le Fujian, et B (*n* = 3) dans le Heilongjiang. Sur les 22 échantillons positifs, 20, 19 et 9 ont été efficacement amplifiés et séquencés respectivement pour les loci *tpi*, *gdh* et *bg*. Sept échantillons ont été génotypés avec succès sur les trois loci, identifiant MLG-B1 (*n* = 1), MLG-B2 (*n* = 1) et MLG-B3 (*n* = 1), MLG-B4 (*n* = 1), MLG- B5 (*n* = 2) et MLG-B6 (*n* = 1) comme les six assemblages MLG B. Cette étude marque la première investigation de *G. duodenalis* chez les ours noirs d’Asie en captivité au Fujian et au Heilongjiang. L’identification des assemblages zoonotiques A et B, ainsi que E, souligne des problèmes potentiels de santé publique.

## Introduction

*Giardia duodenalis*, a major cause of parasite-related diarrheal illness around the world, affects people and many animals, such as amphibians, birds, and mammals [6, 19]. This protozoan parasite leads to around 280 million cases of giardiasis annually in humans, causing diarrhea or other intestinal symptoms, with asymptomatic infections being equally prevalent [8]. Recognizing its impact, the World Health Organization (WHO) classified giardiasis among Neglected Diseases in 2004 [21]. The cysts of *G. duodenalis*, the infection stage, are excreted in feces, facilitating its spread through the fecal-oral route [25]. These cysts can survive in water and other environments, even in the presence of chlorine disinfectants, which accounts for their role in numerous water-borne disease outbreaks [15]. Over the past four decades, at least 132 water-borne outbreaks of giardiasis have been documented [7]. Food-borne outbreaks have also occurred, associated with contaminated food handled by infected food handlers, placing *G. duodenalis* 11th among 24 food-borne parasite species listed by the Food and Agriculture Organization of the United Nations (FAO) [18]. Despite the global focus on *G. duodenalis* due to its capacity to cause outbreaks, treatment options remain limited, with no clinically approved vaccines available [1]. Effectively addressing *G. duodenalis* infections requires a thorough understanding of the sources of infection and transmission dynamics.

The application of molecular diagnostics to investigate *G. duodenalis* infections presents a significant advancement in understanding the epidemiology of this parasite. Molecular typing tools focusing on specific parasitic genes, such as the small subunit ribosomal RNA (*SSU rRNA*), glutamate dehydrogenase (*gdh*), triosephosphate isomerase (*tpi*), and β-giardin (*bg*), are crucial for identifying *G. duodenalis* assemblages [28]. Use of these tools provides extensive information regarding the spread and possibility for transmission to humans of *G. duodenalis* assemblages among different animal hosts. Molecular analyses have categorized *G. duodenalis* into assemblages A to H, all found in mammals but with distinct host distributions [11]. Humans and a variety of other mammals are infected by Assemblages A and B, demonstrating a variety of hosts and pandemic transmission capability [6, 19]. Conversely, Assemblages such as C, D, E, F, G, and H exhibit greater host specificity and more constrained host ranges, with C and D primarily infecting canines, E found in cloven-hoofed domestic mammals, and F, G, and H prevalent in cats, rodents, and marine pinnipeds, correspondingly [11, 16, 19]. Notably, there have been documented cases of C, D, E, and F assemblage infection in humans, especially among children and immunocompromized individuals, underscoring the zoonotic transmission role in human giardiasis epidemiology [6, 9, 11, 20, 22]. Understanding the distribution of *G. duodenalis* assemblages in different hosts is crucial for addressing giardia outbreaks effectively.

Illnesses associated with *G. duodenalis* have increasingly been verified in both captive and free-ranging wildlife through molecular techniques [19]. Unlike *Cryptosporidium*, which shows in species that are host-adapted and genotypes predominating in wild animals, zoonotic *G. duodenalis* Assemblages A and B are frequently identified in numerous studies, indicating that animals in the wild may act as significant reservoirs for human illnesses [11, 19, 20]. Despite this, genetic proof directly associating *G. duodenalis* in wild animals with human giardiasis remains limited. Outdoor enthusiasts and caretakers of captive wildlife, who may come into contact with animal excreta, face potential transmission risks from wildlife to humans [19]. Bears, including grizzly bears in Canada, Sun bears and brown bears in Croatia, brown bears, American black bears and Andean bears in Peru, and polar bears in Arctic Alaska, have all been reported to carry *G. duodenalis* [3, 16, 17, 26]. This evidence suggests that bears could play a crucial part in the spread of *G. duodenalis* and warrant attention. Therefore, routine monitoring and genetic recognition of *Giardia* species/assemblages in these animals are critical for elucidating transmission dynamics and assessing the role of wildlife reservoirs in spreading infections to humans.

In China, an estimated 28.5 million cases of giardiasis occur annually, most of which remain unreported [10]. Recent findings have identified *G. duodenalis* in various animals, emphasizing farm animals (pigs, sheep, and cattle) and pets (dogs and cats) [20]. Asiatic black bears, valued for their economic and medicinal importance, are extensively farmed and also prevalent in zoos for commercial and esthetic purposes. As of now, there are no documented known *G. duodenalis* infections in bears within China. This study investigates the incidence and assemblage dissemination of *G. duodenalis* in bears from Heilongjiang and Fujian Provinces, using multilocus genotyping (MLG) analysis of the *SSU-rRNA, tpi, gdh,* and *bg* genes. The objective is to expand understanding of *G. duodenalis* occurrence, prevalence, and assemblage distribution in bear populations, thereby enhancing public health knowledge and aiding in the development of preventive strategies.

## Methods

### Ethical declaration

The Wenzhou Medical University Research Ethics Committee and the Animal Ethics Committee gave approval for the study design. The owners or managers of the animals gave their express permission for fecal samples to be taken, guaranteeing that no animals suffered injury or discomfort while the samples were being taken.

### Collection of fecal specimens

About 50 g of fresh feces were removed from each of the 218 Asiatic black bears between May 2015 and December 2017. Of these bears, 36 were in a zoo in Heilongjiang Province, and 182 were on a farm in Fujian Province, China (Table 1). Fresh feces samples were gathered from the ground immediately after excretion utilizing sterilized disposable latex gloves, stored in marked sterile bags, and taken to the lab. The samples were kept at 4 °C and processed within 12 h of being collected.

**Table 1:** Prevalence and assemblage of *G. duodenalis* at the *SSU rRNA*, *bg*, *gdh* and *tpi* genes in bears according to province, age, gender, and feeding mode.

Group	Positive no./Examined no. (%)	*G. duodenalis* assemblage				*p* value
		*SSU rRNA* (n)	*bg* (n)	*gdh* (n)	*tpi* (n)	
Age (year)						*p* = 0.019
<3	4/29 (13.8)	B (4)	B (4)	B (4)	B (1)	
3–5	17/122 (13.9)	A (1); B (14), E (2)	A (1); B (14)	B (14)	B (5), E (2)	
>5	1/67 (1.5)	B (1)	B (1)	B (1)	B (1)	
Feeding mode						*p* = 0.002
Alone	6/128 (4.9)	A (1); B (3), E (2)	A (1); B (3)	B (3)	B (1), E (2)	
Group	16/90 (17.8)	B (16)	B (16)	B (16)	B (6)	
Gender						*p* = 0.099
Male	17/133 (12.8)	A (1); B (14), E (2)	A (1); B (14)	B (14)	B (4), E (2)	
Female	5/85 (5.9)	B (5)	B (5)	B (5)	B (3)	
Provinces						*p* = 0.147
Fujian	19/182 (10.4)	A (1), B (16), E (2)	A (1), B (16)	B (16)	B (6), E (2)	
Heilongjiang	3/36 (8.3)	B (3)	B (3)	B (3)	B (1)	
Total	22/218 (10.1)	B (19), E (2), A (1)	B (19), A (1)	B (19)	B (7), E (2)	

### Data logs

Data on the Asiatic black bears, comprising sex, age, and fecal characteristics, were recorded by the trainers and organized into a database using MS Excel 2007. In Fujian Province, 128 bears were divided into three categories: 17 were under 3 years old, 122 were aged between 3 and 5 years, and 43 were older than 5 years. The gender distribution included 118 males and 64 females, with 92 bears raised individually and 90 in groups. There were 12 bears under 3 years old and 24 bears over 5 years old in Heilongjiang Province. In Heilongjiang, each of the 36 bears was reared separately, comprising 15 males and 21 females. When the samples were being collected, no signs of illness were observed in any animal.

### Extraction of DNA

Feces samples were centrifuged at 1500×*g* for 10 min after passing using an 8.0-cm-diameter strainer with 45 μm pores. Following the supplier’s instructions, genomic DNA was subsequently extracted from 200 mg of the concentrated feces sample through a QIAamp DNA Stool Mini Kit (QIAGEN, Hilden, Germany). The isolated DNA was kept cold for use in PCR testing later on.

### Amplification by PCR

To determine whether *G. duodenalis* was present in the DNA samples, nested PCR was used to target the *SSU rRNA* gene. The *bg*, *tpi*, and *gdh* genes were the main focus of MLG analysis performed on samples that tested positive for the *SSU rRNA* gene by nested PCR. The primers and PCR reaction conditions for amplifying the *SSU rRNA* (~290 bp), *tpi* (~530 bp), *bg* (~510 bp), and *gdh* (~530 bp) genes followed the methodologies illustrated by Appelbee et al., Lalle et al., Sulaiman et al., and Cacciò et al., respectively [2, 5, 13, 23]. All PCR amplifications used TaKaRa Taq DNA Polymerase (TaKaRa Bio Inc., Tokyo, Japan). DNA from a brown rat-derived *G. duodenalis* assemblage G acted as positive controls in assays of PCR for the *SSU rRNA, bg, gdh*, and *tpi* genes to ensure effective amplification and verify DNase absence. DNase-free water served as a negative control in each assay of PCR to monitor for contamination. The 1.5% agarose gel electrophoresis of the PCR results was followed by a Gel Doc EZ UV-gel imaging system (Bio-Rad Inc., Hercules, CA, USA) for graphical representation, which was further improved by a GelRed (Biotium Inc., Hayward, CA, USA) staining process.

### Analysis and sequencing of nucleotides

PCR amplicons generated from positive nested PCR reactions targeting *G. duodenalis* were directed to Sangon Biotech Co., Ltd. (Shanghai, China) for purification and sequencing. The sequencing utilized a Big Dye Terminator v3.1 Cycle Sequencing Kit (Applied Biosystems, Waltham, MA, USA) on an ABI PRISM 3730 XL DNA Analyzer. To ensure sequence accuracy, bidirectional sequencing was performed, with additional PCR products sequenced as required. The sequences were edited using DNASTAR Lasergene EditSeq v7.1.0 (http://www.dnastar.com/), and Clustal X v2.1 (http://www.clustal.org/) was used to align the altered sequences with reference sequences that were downloaded from GenBank.

### Examining data statistically

The prevalence of *G. duodenalis* and its association with risk factors such as geographical location, age, gender, and feeding mode were analyzed using the chi-square (*χ*^2^) test in SPSS software version 22.0 (SPSS Inc., Chicago, IL, USA). Statistical significance was determined at a *p*-value threshold of less than 0.05.

### Nucleotide sequence accession numbers

The nucleotide sequences of *G. duodenalis* obtained in the present study were deposited in the GenBank database under accession numbers PP356052 to PP356057 for the *SSU-rRNA* gene, PP388936 to PP388938 for the *tpi* gene, PP388939 to PP388942 for the *gdh* gene, and PP388943 to PP388948 for the *bg* gene.

## Results

### Infection rates of *G. duodenalis*

The nesting PCR targeting the *SSU-r-RNA* gene revealed *Giardia* sp. presence in 22 out of 218 (10.1%) samples, with 19 (10.4%) from the farm in Fujian and 3 (8.3%) from the zoo in Heilongjiang (Table 1). The prevalence difference between the farm and zoo was not statistically significant (*p* = 0.147). Among age groups, *G. duodenalis* occurrence was 13.8% (4/29) in bears under 3 years, 13.9% (17/122) in those aged 3–5 years, and 1.5% (1/67) in those over 5 years, with the variance being significant (*χ*^2^ = 7.884, df = 2, *p* = 0.019). Additionally, the prevalence in males (12.8%; 17/133) was higher than in females (5.9%; 5/85), although the difference was not significant (*p* = 0.099). The occurrence in group-fed bears was 17.8% (16/90), significantly higher than in individually-fed bears (4.9%; 6/128) (*χ*^2^ = 9.98, *p* = 0.002).

### Molecular characterization

#### Identification of *Giardia* assemblages

Three assemblages (A, B, and E) of *G. duodenalis* were found in the bears using sequencing of the *SSU rRNA* gene. Assemblage B was predominant, representing 86.4% (19/22) of the cases, and was found in animals from the provinces of Fujian and Heilongjiang. Isolates belonging to assemblage A (4.5%, 1/22) and assemblage E (9.0%, 2/22) were exclusively found in bears from Fujian Province. Regarding age distribution, assemblage B was present across all age groups, while assemblages A and E were noticed solely in animals aged 3–5 years. Assemblages A and E were recognized only in solitary male bears. In contrast, assemblage B was observed in bears from both solitary and group feeding modes and in both genders (Table 1).

#### Genetic diversity of *Giardia* assemblage at the *SSU-rRNA* locus

Among the 19 SSU rRNA sequences of assemblage B, four haplotypes were recognized, labeled *ssuB1* to *ssuB4*. The *ssuB1* haplotype (*n* = 13) matched 100% with a previously identified *G. duodenalis* assemblage B sequence (HQ616612) from *Lemur catta* in Spain, alongside 91 other sequences in GenBank. The remaining six SSU rRNA sequences of assemblage B were novel, showing only minor variations from the ssub1 sequence. Specifically, *ssuB2* (*n* = 4), *ssuB3* (*n* = 1), and *ssuB4* (*n* = 1) sequences each had a single nucleotide difference at positions 6 (C to T), 7 (A to G), and 138 (G to A) relative to *ssuB1*, respectively. The SSU sequence of assemblage A matched a sequence that had been reported from *Lynx pardinus* from Spain and another 48 sequences stored in GenBank. Both SSU sequences of assemblage E were previously described, demonstrating homology to a *G. duodenalis* assemblage E sequence discovered in sheep in China and another 15 sequences stored in GenBank (Table 2).

**Table 2:** Similarity analysis of *SSU-rRNA*, *bg*, *gdh*, and *tpi* sequences within *G. duodenalis* assemblage A, assemblage B, and assemblage E.

Genes, subassemblages (n)		Accession number(s)	Identities/Nucleotide positions	Ref accession numbers in host from country
*SSU-rRNA*				
	*ssuB1* (13)	PP356053	100%/–	HQ616612^a^ from *Lemur catta* in Spain
	*ssuB2* (4)	PP356052	99.59%/(C to T at position 6)	
	*ssuB3* (1)	PP356054	99.59%/(A to G at position 7)	
	*ssuB4* (1)	PP356055	99.59%/(G to A at position 138)	
	*ssuA* (1)	PP356056	100%/–	OR916207^b^ in *Lynx pardinus* from Spain
	*ssuE* (2)	PP356057	100%/–	OR359372^c^ in sheep from China
*bg*				
	*bgB1* (11)	PP388943	100%/–	OM115991 from humans in Iran
	*bgB2* (1)	PP388944	100%/–	OM115989 from humans in Iran
	*bgB3* (3)	PP388945	99.79%/(A delete at position 10)	KJ888976 from *Macaca mulatta* in China
	*bgB4* (3)	PP388946	99.79%/(A delete at position 10)	MT487587 from a masked palm civet in China
	*bgB5* (1)	PP388947	99.79%/(A delete at position 10)	MG736242 from a human in Egypt
	*bgA* (1)	PP388948	99.57%/(A to G at position 18 and G to A at position 366)	ON168867 from a pig in China
*gdh*				
	*gdhB1* (8)	PP388939	100%/–	EU834844 in human from Belgium
	*gdhB2* (6)	PP388940	99.60%/(T to G at position 427 and A to G at position 463)	KF679732 in a rhesus macaque from China
	*gdhB3* (3)	PP388941	99.80%/(G to A at position 364)	MK952603 in a golden monkey from China
	*gdhB4* (2)	PP388942	99.80%/(C to T at position 145)	MK952600 in a *Ring-tailed Lemur* from China
*tpi*				
	*tpiB* (7)	PP388936	99.62%/(C to T at position 12, and C to T at position 490)	MG736281 from a human in Egypt
			99.62%/(G to T at position 12, and A to G at position 24)	KX085491 from a human in Brazil
	*tpiE*1 (1)	PP388938	99.81% (C to T at position 101)	MH079446 from cattle from China
	*tpiE*2 (1)	PP388937	99.81% (G to A at position138)	MH079446 from cattle from China

^a^Another 91 sequences stored in GenBank.

^b^Another 48 sequences stored in GenBank.

^c^Another 15 sequences stored in GenBank.

#### Genotyping at the triose phosphate isomerase locus

At the *tpi* locus, sequencing successfully analyzed only 9 of the 22 isolates (40.9%), with none previously described. Seven of these sequences were identical (*tpiB*) and shared 99.62% similarity with the assemblage B sequences MG736281 and KX085491 from humans in Egypt and Brazil, respectively, with nucleotide substitutions at positions 12 (C to T; G to T) and 490/24 (C to T; A to G). The remaining two sequences, designated *tpiE1* and *tpiE2*, exhibited 99.81% similarity to the assemblage E sequence MH079446 from cattle in China, varying between positions 138 (G to A) and 101 (C to T), accordingly, by a single nucleotide (Table 2).

#### Genotyping at the beta-giardin locus

Sequencing successfully analyzed 20 of the 22 samples (90.9%) at the *bg* locus, but no sequences were obtained for the two assemblage E samples. BLAST analysis identified six haplotypes, designated *bgB1* to *bgB5* for Assemblage B and *bgA* for Assemblage A. Haplotypes *bgB1* (*n* = 11) and *bgB2* were identical to subassemblage BIII isolates (OM115991 and OM115989) from humans in Iran. Haplotypes *bgB3* to *bgB5* each displayed a single base deletion compared to sequences KJ888976 from *Macaca mulatta* in China, MT487587 from a masked palm civet in China, and MG736242 from a human in Egypt, respectively. The *bgA* haplotype showed 99.57% similarity to sequence ON168867 from a pig in China, with two base transitions at positions 18 (A to G) and 366 (G to A) (Table 2).

#### Glutamate dehydrogenase locus genotyping

Clear amplification products were derived from 19 of the 22 isolates (86.4%) at the *gdh* locus using generic primers. Four haplotypes, *gdhB1* to *gdhB4*, were identified within Assemblage B. The *gdhB1* sequence, representing eight samples, matched 100% with the sequence EU834844 from a human in Belgium. Haplotypes *gdhB2* to *gdhB4* are novel, with nucleotide transitions observed between *gdhB1* and *gdhB2* at loci 104 (T to C) and 365 (G to A), between *gdhB1* and *gdhB3* at sites 104 (T to C), 146 (T to C), and 299 (T to C), and between *gdhB1* and *gdhB4* at sites 104 (T to C), 146 (T to C), 173 (T to C), 299 (T to C), 308 (C to T), and 463 (A to G). No sequences were obtained for the three samples belonging to Assemblage A or E at the *gdh* locus (Table 2).

#### MLG examination of bear *Giardia* isolates

MLG-B1 (*n* = 1), MLG-B2 (*n* = 1), and MLG-B3 (*n* = 1), MLG-B4 (*n* = 1), MLG-B5 (*n* = 2), and MLG-B6 (*n* = 1) were the names given to the 7 sequences out of the 22 bear specimens that tested positive for *G. duodenalis* (Table 3).

**Table 3:** Multilocus characterization of *G. duodenalis* isolates based on the *SSU rRNA*, *bg*, *gdh*, and *tpi* genes.

Sample ID	*SSU rRNA*	Sub-assemblage			MLG
		*bg*	*gdh*	*tpi*	
FJB49	*ssuB1*	*bgB1*	*gdhB1*	PN	/
FJB55	*ssuB2*	*bgB3*	*gdhB1*	PN	/
FJB83	*ssuB2*	*bgB2*	*gdhB1*	PN	/
FJB91	*ssuB1*	*bgB3*	*gdhB2*	*tpiB*	MLG-1
FJB95	*ssuB1*	*bgB1*	*gdhB3*	PN	/
FJB96	*ssuB1*	*bgB1*	*gdhB3*	*tpiB*	MLG-2
FJB101	*ssuB1*	*bgB4*	*gdhB1*	*tpiB*	MLG-3
FJB103	*ssuB1*	*bgB4*	*gdhB4*	*tpiB*	MLG-4
FJB106	*ssuB1*	*bgB3*	*gdhB2*	PN	/
FJB111	*ssuB1*	*bgB1*	*gdhB1*	*tpiB*	MLG-5
FJB113	*ssuB1*	*bgB4*	*gdhB2*	*tpiB*	MLG-6
FJB124	*ssuB2*	*bgB1*	*gdhB2*	PN	/
FJB126	*ssuB1*	*bgB1*	*gdhB3*	PN	/
FJB149	*ssuB1*	*bgB1*	*gdhB2*	PN	/
FJB158	*ssuB1*	*bgB1*	*gdhB2*	PN	/
FJB163	*ssuA*	*bgA*	*PN*	PN	/
FJB169	*ssuB2*	*bgB5*	*gdhB4*	PN	/
FJB171	*ssuE*	PN	PN	*tpiE*	/
FJB177	*ssuE*	PN	PN	*tpiE*	/
HLJB4	*ssuB1*	*bgB1*	*gdhB1*	*tpiB*	MLG-5
HLJB5	*ssuB3*	*bgB1*	*gdhB1*	*PN*	/
HLJB11	*ssuB4*	*bgB1*	*gdhB1*	*PN*	/

PN: PCR negative.

## Discussion

This study represents, as far as we are aware, the initial documentation of *G. duodenalis* in Chinese bears uncovering an overall prevalence of 10.1%, with 10.4% in a farm in Fujian and 8.3% in a zoo in Heilongjiang. While reports of Ursidae infections with *G. duodenalis* exist, only one comprehensive survey from Croatia, revealing an infection rate of 4.3%, has been published on bears [16]. A direct comparison with other regions is challenging due to the limited comprehensive data from previous studies. Several factors, including the sample size, examination methods, immune status of the animals, and seasonal variations, influence the infection rates within specific hosts. Notably, bears younger than 5 years exhibited significantly higher infection rates than those older than 5 years, suggesting increased susceptibility to *G. duodenalis* infection among younger bears. These findings are consistent with research conducted on other animal species, such as cattle, cats, and dogs [4, 24]. An exhaustive examination and systematic review of stool sample prevalence studies revealed that *G. duodenalis* is more prevalent between young dogs and cats, with similar observations noted in calves within the cattle population [4, 24]. This study found a lower prevalence of *G. duodenalis* in female bears than in male bears, although the relationship between gender and infection rate remains unclear. While gender may not directly influence *G. duodenalis* infection rates, offspring of infected mothers, particularly during breastfeeding, have a higher likelihood of transmission, highlighting increased susceptibility among children due to close maternal contact. Feeding methods also influence the infection rate, with group-fed animals exhibiting a *G. duodenalis* prevalence of 17.8%, 3.6 times higher than the 4.9% observed in individually-fed animals. This disparity is attributed to the greater interaction among animals during group feeding, facilitating the spread of infection, thereby underlining the importance of hygiene practices in controlling *G. duodenalis* infections. Geographical variations in prevalence were observed, with animals in Fujian showing higher rates than those from Heilongjiang, although differences were not statistically significant. The smaller sample size from Heilongjiang than Fujian may have affected the observed prevalence variations. Hence, larger-scale surveys are recommended to explore the relationship between *G. duodenalis* prevalence and related risk variables further.

Analysis across four loci (*SSU rRNA, gdh, bg,* and *tpi*) confirmed the existence of three *G. duodenalis* assemblages (A, B, and E) in the black bears examined. Assemblage B was significantly prevalent, aligning with findings in Sun bears and brown bears in Croatia, where this assemblage predominates [16, 26]. This genotype is prevalent in humans and various animal species in China, including non-human primates, pigs, cattle, sheep, horses, and other wild animals [14, 27]. Assemblage A is also common in China among both humans and non-human primates as well as pigs, livestock, and sheep, and was detected in one bear, indicating a potentially broader host range [14, 27]. The existence of assemblages A and B underscores the potential of bears as reservoirs for human infections, highlighting the need for further research on interspecies transmission.

In the present research, assemblage E of *G. duodenalis* was detected alongside assemblages A and B. Assemblage E, predominantly found in livestock and previously regarded as ruminant-specific, has evidence suggesting its zoonotic transmission potential between humans and animals [11]. It has been identified in 54 human cases globally, mainly in rural areas, with individuals presenting gastrointestinal symptoms, with or without diarrhea [10]. Molecular analyses have revealed identical genetic types in human cases and livestock (cattle and sheep), indicating a strong likelihood of zoonotic transmission of Assemblage E [9–11, 30]. The pathway for Assemblage E’s introduction to bears remains unclear, although water or food contamination by domestic animals is the most probable route. While Assemblage E is uncommon in wild animals, cross-transmission between wild and domestic animals has been documented, such as in red colobus monkeys from Uganda, suggesting cross-transmission from cattle [12].

The *SSU rRNA* locus exhibited the highest amplification rate and was the most conserved among the four studied loci (*SSU rRNA, tpi, gdh, and bg*). In contrast, the *tpi* locus showed the lowest amplification rate, with positive samples accounting for only 40.9% (9/22). The *gdh* and *bg* loci demonstrated high amplification rates and significant genetic variation. This study indicates that the amplification success of the *bg, gdh*, and *tpi* genes correlates with the assemblage type, with Assemblages A and B more frequently amplified at the *bg* and *gdh* genes, whereas Assemblage E showed greater success at the *tpi* locus. Similar observations regarding differences in amplification rates across gene loci have been reported in previous research. For instance, Xu et al. investigated the *bg*, *gdh*, and *tpi* genes and yielded 40, 34, and 59 sequences from 129 *G. duodenalis*-positive isolates, respectively, in donkeys in Xinjiang, China [29]. Out of 195 positive samples, only 11 and 6 samples, accordingly, were found to have *G. duodenalis* at the *tpi* and *gdh* loci in research conducted in pigs in Fujian Province, China [31]. The observed low amplification rates for the *gdh, bg*, and *tpi* loci highlight the need for more sensitive typing techniques to enable comprehensive genetic characterization of these isolates.

MLG analysis on three genes identified six novel MLGs (MLG-B1, MLG-B2, MLG-B3, MLG-B4, MLG-B5, and MLG-B6) within assemblage B, indicating the potential existence of unique subassemblages B in *G. duodenalis* found in bears. Although no MLGs for assemblages A and E were determined due to unsuccessful amplification at all four loci, genetic diversity was evident, as indicated by novel sequences *bgA*, *tpiE1*, and *tpiE2*. This finding underscores the need for further investigation to elucidate the specific mechanisms involved, given the absence of descriptions on MLG typing of *G. duodenalis* in bears. Future studies are needed to verify the presence of assemblage A and E infections in bears.

## Conclusions

This report provides an initial examination of the occurrence of *G. duodenalis* in black bears farmed and housed in zoos across two provinces in China, detailing the features of the assemblages and assessing the potential for zoonotic transmission. The characteristics of assemblages A, B, and E in bears suggest the capability of these animals to transmit giardiasis to humans and domestic animals. Moreover, detecting novel sequences highlights the possibility of unique regional or host-specific correlations, underscoring the need for further investigation to fully comprehend the zoonotic potency and spread dynamics of *G. duodenalis* within bear populations.
